# Effect of preoperative virtual reality cartoon viewing on postoperative pain and anxiety in children undergoing tonsillectomy and adenoidectomy: A randomized controlled trial

**DOI:** 10.1371/journal.pone.0331793

**Published:** 2025-09-11

**Authors:** Seda Kırhallı Mersin, Berna Dizer, Arzu Tuna

**Affiliations:** 1 Uşak University Training and Research Hospital, Uşak, Turkey; 2 Vocational School of Health Services, Operation Room Services Department, Izmir Tınaztepe University, Izmir, Turkey; 3 Balıkesir University Faculty of Health Sciences, Surgical Nursing Department, Balıkesir, Turkey; Universiti Malaya Fakulti Perubatan: University of Malaya Faculty of Medicine, MALAYSIA

## Abstract

**Objective:**

Surgery causes anxiety in children and negatively affects postoperative pain control. Various distraction methods, such as virtual reality (VR), have been shown to reduce anxiety levels and improve surgical outcomes. This study aimed to determine the effect of watching cartoons through a VR headset before surgery on systolic blood pressure, postoperative pain, and anxiety levels as primary, secondary, and tertiary outcomes, respectively, in children aged 7–12 years undergoing tonsillectomy and adenoidectomy.

**Methods:**

This randomized controlled experimental study was conducted at a tertiary hospital between November 10, 2023, and June 1, 2024, among 102 children scheduled for tonsillectomy and adenoidectomy, who were randomly divided into an experimental group (n = 51; VR intervention) and a control group (n = 51; no intervention). The primary outcomes were anxiety levels measured using the Perioperative Multidimensional Anxiety Scale for Children and postoperative pain evaluated using the Visual Analog Scale. Sociodemographic characteristics and vital signs were also assessed.

**Results:**

Systolic blood pressure values were significantly lower in the experimental group at than in the control group at all time points (p < 0.05). Postoperative pain values were lower in the experimental group (3.35 ± 1.43 vs. 6.53 ± 1.36, p < 0.05), with similar results observed 8 h post-surgery (1.29 ± 1.08 vs. 6.57 ± 1.17, p < 0.05). Anxiety values were also significantly lower in the experimental group (24.12 ± 11.17 vs. 69.41 ± 12.56, p < 0.05), with similar results observed 8 h post-surgery (12.35 ± 10.50 vs. 67.0 ± 11.37, p < 0.05).

**Conclusion:**

VR technology, particularly through watching the *Shrek* cartoon, significantly reduced systolic blood pressure, pain, and anxiety levels in children undergoing tonsillectomy and adenoidectomy. Thus, VR could be an effective noninvasive tool for managing pain and anxiety in pediatric patients during the preoperative and postoperative periods.

**Trial registration:**

ClinicalTrials.gov (NCT06763276).

## Introduction

Tonsillectomy and adenoidectomy are among the most frequently performed pediatric surgeries, with over 250,000 cases reported annually in the United States, representing a significant portion of the 6–8% of all surgeries conducted in children [1]. These procedures are commonly indicated for conditions such as recurrent tonsillitis, adenoiditis, and obstructive sleep apnea, which significantly affect pediatric health and quality of life [1]. Despite their high frequency, outcomes and complication rates vary depending on surgical techniques and patient characteristics, highlighting the need for continued research for improvement [1]. In addition to the physiological outcomes of these surgeries, their emotional impact on children is profound, as children may experience considerable preoperative anxiety and stress, adversely affecting their postoperative recovery [2].

Various health issues can arise during childhood, sometimes necessitating hospitalization and surgery, which can be a source of fear and anxiety in both children and parents. Preoperative anxiety significantly impacts postoperative pain and recovery. Physiologically, preoperative anxiety can increase stress hormone levels, thereby intensifying pain perception, prolonging anesthesia induction, and delaying recovery [2]. Kain et al. [3] found that children with high preoperative anxiety levels required more postoperative analgesics, such as paracetamol and codeine, experienced longer recovery times, and necessitated higher doses of propofol for anesthesia induction [2], underscoring the importance of managing preoperative anxiety to improve surgical outcomes.

Children, who may not fully understand concepts such as illness and death, often find hospitalization and surgery traumatic [4]. Hospital can also be stressful and traumatic for parents, as families are mainly responsible for helping children navigate such a situation. A child sensing parents’ anxiety may fear the surgical process. Parents’ calm and supportive attitude toward their children before surgery can help reduce the children’s anxiety and fear [5]. Psychological support provided by both the family and nursing staff plays a crucial role in managing the child’s pain and anxiety [6].

Surgical interventions are often performed to alleviate pain; however, postoperative pain can occur [7]. Anxiety and pain negatively affect post-surgery comfort and recovery in children. Nurses and medical treatments are crucial in reducing children’s anxiety and pain levels [8]. Owing to their lack of understanding of operating rooms and surgical procedures, children often fear pain. Factors such as the child’s age, nature and urgency of the surgery, and hospital stay length can aggravate this fear [9].

İnal and Canbulat [10] reported that children’s anxiety levels decreased when passive visual stimuli aimed at general relaxation were provided using virtual reality (VR) glasses before surgery. During the preoperative period, nurses should provide personalized care to children and their families, considering their individual characteristics [6,11]. Various activities, including playing music, inflating balloons, drawing, or watching cartoons through VR glasses, can be employed to distract children [6,10,12,13].

VR, audiovisual distractions, and behavioral interventions are methods used to reduce preoperative anxiety. Research has shown that VR interventions are effective in reducing preoperative anxiety and postoperative pain, even in children undergoing tonsillectomy and adenoidectomy [14,15].

Surgery is a painful procedure, and understanding pain levels in children is challenging, as their ability to express themselves is not as developed as that of adults. Moreover, anxiety in children regarding surgery raises their pain levels [16]. Tonsillectomy and adenoidectomy can significantly increase anxiety and pain in children [17].

Various distraction methods, including the use of VR, have been employed during invasive procedures such as peripheral cannulation, intramuscular injection, venous blood collection, vaccination, and dental procedures [7,10,18,19]. However, no studies have been conducted to specifically examine the effects of pre- and postoperative use of VR on fear and anxiety in children undergoing tonsillectomy and adenoidectomy. In addition, only a few studies have been conducted on the use of VR in preoperative and postoperative settings, indicating the need for further research.

Considering the preoperative psychological and emotional needs of children, it is important to investigate whether showing cartoons using VR glasses provides additional benefits. Filling this knowledge gap could offer valuable insights into improving care standards in pediatric surgery and reducing the stress associated with such procedures.

This study aimed to explore the potential benefits of VR technology in pediatric surgery and to guide future practices. Specifically, this study was conducted to investigate the effect of watching cartoons via VR glasses before surgery on pain and anxiety levels in children aged 7–12 years undergoing tonsillectomy and adenoidectomy. The findings suggest that VR could be incorporated into routine pediatric surgical care as a simple yet effective noninvasive approach to reduce pain and anxiety, ultimately enhancing patient outcomes.

## Materials and methods

### Study design and setting

This study was registered at ClinicalTrials.gov (Registration Number: NCT06763276; Date: 2024-07-26 and Enrolment no: 102). This trial was retrospectively registered because the requirement for prospective registration was not initially recognized. Ethical approvals and institutional permissions were obtained before participant recruitment, but due to an administrative oversight, the trial registration process was initiated after recruitment had begun. Once the requirement was acknowledged, immediate steps were taken to complete the registration. The trial protocol is included as a Supporting Information file. The authors confirm that all ongoing and related trials for this drug/intervention are registered.

Participants were recruited into this study from November 10, 2023, to June 4, 2024, at the Ear, Nose, and Throat unit of Uşak University Education and Research Hospital. Patient follow-up continued until July 13, 2024.

### Outcome measures and Randomization

The primary, secondary, and tertiary outcomes included systolic blood pressure, postoperative pain, and anxiety level, respectively. Randomization was performed using a computerized random number generator by an independent researcher who was not involved in data collection. Participants were randomly assigned to experimental and control groups and coded as 1 and 2, respectively.

The study was conducted at the Ear, Nose, and Throat unit of Uşak University Education and Research Hospital, which has a total of 24 patient beds. Preoperatively, the children were escorted to the operating room by staff and primary nurses. Primary nurses are those who continue to care for the children and administer treatments as instructed by doctors postoperatively. Primary nurses play a key role in meeting the physical and emotional needs of children before and after surgery by working closely with their families to minimize surgical stress. Preoperative preparation includes informing and calming children about the surgical process, while postoperative care involves monitoring vital signs, managing pain, and assessing anxiety. The primary, secondary, and tertiary outcomes included systolic blood pressure, postoperative pain, and anxiety level, respectively, each measured at three time points. Meticulous process management was implemented to enhance the reliability and validity of this study.

The CONSORT checklist was applied in the study [20] (https://www.equator-network.org/).

### Participants

#### Inclusion and exclusion criteria.

Children eligible for the study were required to be between the ages of 7 and 12 years and able to understand the pain and anxiety scales; have communication skills; and have no mental, physical, or chronic illnesses. In addition, they needed to have no issues with vision, hearing, or speech, and the consent of both the children and their families was necessitated. Children who did not want to wear glasses, had to wear regular glasses continuously, had hearing problems, or did not want to watch the *Shrek* cartoon were excluded from the study.

#### Study population and sample.

The study population comprised children aged 7–12 years scheduled for tonsillectomy and adenoidectomy at Uşak University Education and Research Hospital’s Ear, Nose, and Throat unit. The study initially included 108 children. However, six children (three from each group) were excluded from the study for reasons such as not wanting to watch the film or wanting to remove their VR glasses. Finally, 102 children were included in this study.

**Fig 1:**
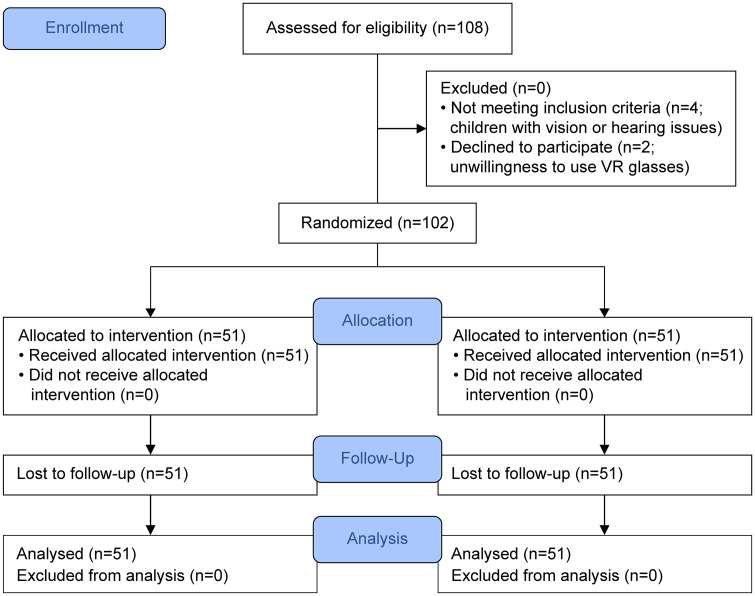
CONSORT chart.

*G* Power analysis was conducted based on an F-test value for repeated-measures analysis of variance, within factors, with an effect size f of 0.25, a critical F of 2.69372, a degree of freedom of 3.000, a power of 80%, and a significance level (α) of 0.05. The sample size was calculated based on the primary outcome of systolic blood pressure, measured at three time points. The experimental and control groups consisted of 51 children each. Random assignment was performed using a computer-generated randomization list to prevent interactions between the experimental and control groups, ensuring that the order of patient treatment did not affect group allocation. The randomization process was conducted by an independent researcher who was not involved in data collection or analysis to minimize bias. Although the sample size was calculated based on repeated-measures analysis of variance assumptions, normality tests indicated a non-normal distribution of the study variables; therefore, nonparametric tests (Mann–Whitney U and Friedman) were appropriately used in the final analysis. Fig 1 shows the CONSORT chart used in this study.

### Study flow

The study sample comprised 102 children who met the inclusion criteria and agreed to participate before undergoing tonsillectomy and adenoidectomy. Before the trial started, all patients received the standard medical treatment and nursing interventions, including routine preoperative procedures such as administration of preoperative medications, monitoring of vital signs, and provision of information to children and their families about the surgical process, as part of the hospital’s standard care protocols aimed at minimizing preoperative anxiety in pediatric patients.

The steps for the control group were as follows: the children and their parents were introduced to the study, and informed consent was obtained after providing information about the study. Mothers were asked to comfortably care for their children. Vital signs, anxiety, and pain levels were measured before surgery, immediately after the children returned to the ward, and 8 h postoperatively. To ensure ethical fairness, children in the control group were allowed to watch cartoons using VR glasses after all data had been collected.

The steps for the experimental group were as follows. The children and their parents were introduced to the study, informed about its content, and signed consent forms. They were shown how to use the VR glasses. The mothers were asked to comfortably care for their children before surgery. The first 15–20 min of the cartoon were shown to the children before surgery, as it was considered that children’s attention spans might decrease after this time, and they were then sent for the tonsillectomy and adenoidectomy procedures. The remaining part of the cartoon was shown 1 h after surgery, once the children had returned to the ward and were assessed. Vital signs, anxiety, and pain levels were measured before surgery, immediately after the children returned to the ward, and 8 h postoperatively.

Children in the experimental group watched the 3D animation *Shrek* using VR glasses. This movie was chosen because of its short duration, high resolution, and engaging content, making it appealing to children. *Shrek* is a well-known international cartoon that caters to the interests of children aged 7–12 years. The movie was streamed in Turkish on YouTube (https://www.youtube.com/watch?v=2lCAjAccEKg).

The VR glasses used in this study were the BoboVR Z5 model, which features an antireflective lens system. These glasses were compatible with all smartphones with screens between 4.5 and 7 inches and weighed 400 g, making them lightweight and comfortable for children to wear. The glasses were secured to the child’s head with three points of support to ensure stability and disinfected after each use to maintain hygiene. The smartphone was inserted into a detachable compartment in front of the VR glasses. Once secured, the YouTube VR feature was activated to stream the cartoon in 3D. The glasses were obtained via Amazon.

Afterward, sociodemographic data were collected, and the children’s vital signs were monitored. Pain was assessed using the Visual Analog Scale, and anxiety was assessed using the Perioperative Multidimensional Anxiety Scale for Children.

### Data collection tools

Participants voluntarily took part in this study after providing both verbal and written consent and signing an informed consent form. Data collection began on November 10, 2023, and ended on July 4, 2024. Data were collected by a researcher with 5 years of experience in the department using interviews and observation forms. To minimize the risk of bias, the researcher responsible for data collection was blinded to the group assignments of the children.

Both written and verbal informed consent were obtained from all participants before participation in the study. Verbal consent was first documented by the researcher, followed by written informed consent obtained from the participants’ legal representatives (parents or guardians) through signed consent forms. As the study involved minors, written consent was specifically obtained from their parents or legal guardians before their participation. The study was conducted in accordance with the principles of voluntarism and approved by the Uşak University Non-Interventional Clinical Research Ethics Committee (Approval No. 230-230-14).

### Sociodemographic data collection form.

The form was created by the researcher based on a literature review. It included information such as the child’s age, sex, diagnosis, and family medical history. Assessments were conducted before surgery, immediately after surgery, and 8 h post-surgery.

### Vital signs assessment form.

This form was created to measure the patients’ blood pressure, pulse, respiratory saturation, and body temperature before surgery, immediately after surgery, and 8 h post-surgery.

### Visual analog scale.

This scale was developed by Freyd to evaluate pain severity in children [21]. This scale is 10 cm long, with “0” indicating no pain and “10” representing the most severe pain. The children were asked to mark their pain levels using a scale. The Visual Analog Scale is easy to use and has been proven valid and reliable in the literature. Assessments were conducted preoperatively, immediately after surgery, and 8 h postoperatively.

### Perioperative multidimensional anxiety scale.

This scale comprises five visual analog items scored between 0 and 100, each assessing a different aspect of anxiety. The parameters assessed by this tool were as follows: (1) the child’s current level of general anxiety; (2) the child’s level of fear related to the upcoming procedure; (3) the child’s feeling of tension or nervousness; (4) the child’s specific fear of pain from the surgery (this item is only assessed preoperatively); and (5) the child’s concern about something going wrong during the procedure.

This scale was used with permission from Çelik et al. [22]. The Cronbach’s alpha reliability value of the scale was 0.858 for the first measurement (pre-surgery), 0.916 for the second measurement (on the day of surgery), and 0.864 for the third measurement (1 month post-surgery). The total score was used, and validity and reliability studies have been conducted [23]. In this study, a single measurement was made, and the Cronbach’s alpha coefficient for this measurement was 0.845, indicating high internal consistency and reliability.

### Statistical analysis

The data obtained from the study were analyzed using SPSS Statistics for Windows version 25.0. Descriptive statistical methods (frequency, percentage, median, mean, and standard deviation) were used to evaluate the data. A chi-squared analysis was conducted to test the homogeneity of the groups. Nonparametric tests were chosen based on the Kolmogorov–Smirnov test results, which indicated non-normality for all study variables (p < 0.05, Table 1). The Mann–Whitney U test was used to compare two independent groups, while the Friedman test was used for comparisons of more than two related stages. Bonferroni correction was applied to adjust for multiple comparisons, ensuring the family-wise error rate was maintained at the predefined alpha level (p < 0.05). The primary outcome (systolic blood pressure) and the secondary (pain) and tertiary (anxiety) outcomes were analyzed across three time points: before surgery, immediately after surgery, and 8 h post-surgery.

**Table 1:** Normality analysis results of the study variables.

Variables	Measurement Time	Kolmogorov–Smirnov		Status
**Statistic**	p	
Systolic blood pressure (Primary)	Before surgery	0.250	0.000	Not normal
	Post-surgery in the recovery room	0.297	0.000	Not normal
	8 h post-surgery	0.231	0.000	Not normal
Diastolic blood pressure	Before surgery	0.447	0.000	Not normal
	Post-surgery in the recovery room	0.292	0.000	Not normal
	8 h post-surgery	0.276	0.000	Not normal
Pulse	Before surgery	0.172	0.000	Not normal
	Post-surgery in the recovery room	0.179	0.000	Not normal
	8 h post-surgery	0.158	0.000	Not normal
Respiration	Before surgery	0.243	0.000	Not normal
	Post-surgery in the recovery room	0.206	0.000	Not normal
	8 h post-surgery	0.196	0.000	Not normal
Temperature	Before surgery	0.472	0.000	Not normal
	Post-surgery in the recovery room	0.123	0.001	Not normal
	8 h post-surgery	0.156	0.000	Not normal
Pain (Secondary)	Before surgery	0.472	0.000	Not normal
	Post-surgery in the recovery room	0.113	0.003	Not normal
	8 h post-surgery	0.195	0.000	Not normal
Anxiety (Tertiary)	Before surgery	0.119	0.001	Not normal
	Post-surgery in the recovery room	0.158	0.000	Not normal
	8 h post-surgery	0.205	0.000	Not normal

### Ethical considerations

Before collecting data, ethical approval was obtained from the Uşak University Non-Interventional Research Ethics Committee (09.11.2023, decision no. 230-230-14), and institutional permission was obtained from Uşak University Education and Research Hospital (29.08.2023, decision no. 903). The children and their parents voluntarily participated in this study. Written informed consent was obtained from both children and their parents. The necessary permissions were secured for the use of the Perioperative Multidimensional Anxiety Scale for Children. The study was conducted with full adherence to the ethical principles at each stage.

## Results

### Participant characteristics

The demographic characteristics of the participants are presented in Table 2 (S1 Data and S2 Data). No statistically significant differences were observed between the experimental and control groups in terms of age, sex, school status, presence of allergies, chronic diseases, bleeding disorders, heart disease, growth retardation, regular medication use, anticoagulant use, previous hospitalizations, previous surgeries, presence of genetic diseases, complaints such as snoring, throat infections, sleeping with an open mouth, sleep apnea, maternal age, maternal employment status, paternal age, or paternal employment status (p > 0.05). Both groups were homogeneous.

**Table 2:** Distribution of participant demographic characteristics.

Variables		Control Group		Experimental Group		Test Value	p
		n	%	n	%		
Age (±SD, 7.91 ± 1.41)	**Under 8 years**	28	54.9	33	64.7	1.020^**^	0.313
	**8 years and above**	23	45.1	18	35.3		
Sex	**Female**	22	43.1	28	54.9	1.412^**^	0.235
	**Male**	29	56.9	23	45.1		
School status	**Primary school**	47	92.2	47	92.2	0.000^**^	1.000
	**Middle school**	4	7.8	4	7.8		
Presence of allergies	**Yes**	2	3.9	7	13.7	3.047^**^	0.081
	**No**	49	96.1	44	86.3		
Presence of chronic illness	**No**	51	100.0	51	100.0	-	-
Previous surgery	**Yes**	7	13.7	5	9.8	0.378^**^	0.539
	**No**	44	86.3	46	90.2		
Presence of genetic disease	**No**	51	100.0	51	100.0	-	-
Complaints	**Snoring**	20	19.6	20	39.2	5.104^**^	0.161
	**Throat infection**	27	52.9	21	41.2		
	**Sleeping with mouth open**	8	15.7	7	13.7		
	**Sleep apnea**	6	11.8	3	5.9		
Presence of bleeding disorder	**No**	51	100.0	51	100.0	-	-
Presence of heart disease	**No**	51	100.0	51	100.0	-	-
Presence of growth retardation	**No**	51	100.0	51	100.0	-	-
Use of anticoagulant medication	**No**	51	100.0	51	100.0	-	-
Previous hospitalization	**Yes**	9	17.6	4	7.8	2.204^**^	0.138
	**No**	42	82.4	47	92.2		
Regular medication use	**No**	51	100.0	51	100.0	-	-
Mother's age (±SD, 35.07 ± 4.53)	**35 years and under**	23	45.1	27	52.9	0.628^**^	0.428
	**Over 35 years**	28	54.9	24	47.1		
Mother's employment status	**Employed**	31	60.8	32	62.7	0.042^**^	0.839
	**Unemployed**	20	39.2	19	37.3		
Father's age (±SD, 38.01 ± 4.37)	**38 years and under**	23	45.1	26	51.0	0.353^**^	0.552
	**Over 38 years**	28	54.9	25	49.0		
Father's education	**Middle school graduate**	13	25.5	2	3.9		
	**High school graduate**	20	39.2	25	49.0		
	**University graduate**	15	29.4	20	39.2		
Father's employment status	**Employed**	50	98.0	48	94.1	1.041^**^	0.308
	**Unemployed**	1	2.0	3	5.9		
Total		51	100.0	51	100.0		

* p < 0.05,

** Chi-squared test or Fisher’s exact test

The distribution of participants based on surgery-related characteristics is presented in Table 3. Most of the children in both groups expressed preoperative fear, with the rate being significantly higher in the experimental group (86.3% vs. 98%, p < 0.05). Preoperative crying status of the children was similar between the groups (p > 0.05). However, most children reported that their mothers experienced preoperative fear, with the rate being significantly higher in the control group (92.2% vs. 74.5%, p < 0.05). The presence of preoperative fear in the fathers was similar in both groups (p > 0.05).

**Table 3:** Distribution of participants based on surgery-related characteristics.

Variables		Control Group		Experimental Group		Test value	p
		N	%	N	%		
Administration of any medication within the last 6 h before surgery	No	51	100.0	51	100.0	-	-
Any procedure performed within the last 6 h before surgery	No	51	100.0	51	100.0	-	-
Child expressing fear before surgery	Yes	44	86.3	50	98.0	4.883^**^	**0.027** ^*^
	No	7	13.7	1	2.0		
Child crying before surgery	Yes	35	68.6	27	52.9	2.632^**^	0.105
	No	16	31.4	24	47.1		
Child crying duration before surgery (±SD, 15.02 ± 7.18)	Did not cry	16	31.4	24	47.1	18.126^**^	**0.001** ^*^
	Less than 15 min	6	11.8	18	35.3		
	15 min or more	29	56.9	9	19.6		
Presence of mother's fear before surgery	Yes	47	92.2	38	74.5	5.718^**^	**0.017** ^*^
	No	4	7.8	13	25.5		
Presence of father's fear before surgery	Yes	32	62.7	37	72.5	1.120^**^	0.290
	No	19	37.3	14	27.5		
Total		**51**	**100.0**	**51**	**100.0**		

* p < 0.05,

** Chi-squared test or Fisher’s exact test

### Vital signs

Comparisons of vital signs between and within the groups are presented in Table 4. Regarding systolic blood pressure, a statistically significant difference was observed between the groups before surgery, post-surgery in the recovery room, and 8 h after surgery (p < 0.05). The values were consistently higher in the control group than in the experimental group at all three time points. Regarding body temperature, a statistically significant difference was observed between the groups at all three time points (p < 0.05). Preoperative values were higher in the experimental group than in the control group; however, values immediately post-surgery and 8 h post-surgery were lower in the experimental group.

**Table 4:** Comparison of vital signs, pain, and anxiety between and within groups.

Variables	Measurement Time	Control Group			Experimental Group			Test Değeri	p
		Med	$\overset{―}{X}$	SD	Med	$\overset{―}{X}$	SD		
Systolic blood pressure	Before surgery [1]	100.00	102.94	5.76	90.00	93.33	4.76	-6.972^**^	**0.001** ^*^
	Post-surgery in the recovery room [2]	100.00	103.53	6.27	90.00	91.37	3.48	-7.940^**^	**0.001** ^*^
	8 h post-surgery [3]	110.00	104.31	9.22	90.00	92.94	5.40	-6.814^**^	**0.001** ^*^
	Test value	4.186^***^			6.222^***^				
	P	0.123			**0.045** ^*^				
	Bonferroni				**1>2**				
Diastolic blood pressure	Before surgery	60.00	70.59	75.98	60.00	57.65	6.51	-1.685^**^	0.092
	Post-surgery in recovery room	60.00	59.22	6.59	60.00	58.43	8.57	-1.023^**^	0.306
	8 h post-surgery	60.00	61.18	6.53	60.00	58.43	7.31	-1.917^**^	0.055
	Test value	4.000^***^			0.624^***^				
	P	0.135			0.732				
Pulse	Before surgery [1]	88.00	87.92	2.53	88.00	87.37	2.65	-0.666^**^	0.505
	Post-surgery in the recovery room [2]	88.00	88.51	3.48	88.00	88.06	2.65	-0.219^**^	0.827
	8 h post-surgery [3]	88.00	88.33	3.21	89.00	88.55	2.93	-0.957^**^	0.338
	Test value	4.012^***^			15.269^***^				
	P	0.135			**0.000** ^*^				
	Bonferroni				**3>1**				
Respiration	Before surgery [1]	28.00	27.53	1.58	28.00	27.45	2.18	-0.311^**^	0.756
	Post-surgery in the recovery room [2]	28.00	28.04	1.62	28.00	27.84	1.98	-0.526^**^	0.599
	8 h post-surgery [3]	28.00	27.96	1.72	28.00	27.65	1.72	-1.120^**^	0.263
	Test value	6.700^***^			2.085^***^				
	P	**0.035** ^*^			0.353				
	Bonferroni	**2>1, 3>1**							
Temperature	Before surgery [1]	36.60	35.95	4.20	36.30	36.32	0.19	-5.038^**^	**0.001** ^*^
	Post-surgery in the recovery room [2]	36.50	36.48	0.18	36.30	36.32	0.21	-4.062^**^	**0.001** ^*^
	8 h post-surgery [3]	36.50	36.46	0.20	36.40	36.34	0.20	-3.023^**^	**0.001** ^*^
	Test value	7.264^***^			3.591^***^				
	P	**0.026** ^*^			0.166				
	Bonferroni	**2>1**							
Pain	Before surgery [1]	0.00	1.10	1.88	0.00	0.65	1.88	-1.706^**^	0.088
	Post-surgery in the recovery room [2]	6.00	6.53	1.36	3.00	3.35	1.43	-7.770^**^	**0.001** ^*^
	8 h post-surgery [3]	6.00	6.57	1.17	1.00	1.29	1.08	-8.782^**^	**0.001** ^*^
	Test value	87.685^***^			63.578^***^				
	P	**0.001** ^*^			**0.001** ^*^				
	Bonferroni	**2>1, 3>1**			**2>1, 2>3**				
Anxiety	Before surgery [1]	60.00	58.63	21.26	40.00	45.10	17.82	-3.707^**^	**0.001** ^*^
	Post-surgery in the recovery room [2]	70.00	69.41	12.56	20.00	24.12	11.17	-8.674^**^	**0.001** ^*^
	8 h post-surgery [3]	70.00	67.06	11.37	10.00	12.35	10.50	-8.775^**^	**0.001** ^*^
	Test value	19.126^***^			85.318^***^				
	P	**0.001** ^*^			**0.001** ^*^				
	Bonferroni	**2>1, 3>1**			**1>2, 1>3, 2>3**				

* p < 0.05,

** Mann–Whitney U test,

*** Friedman test. Note: Bonferroni correction was applied separately for each outcome (systolic blood pressure, pain, anxiety) to assess within-group differences across the three time points.

In the experimental group, a statistically significant difference in systolic blood pressure values was observed at the three time points (p < 0.05), with preoperative values being higher than post-surgery values. Additionally, a statistically significant difference in pulse values was observed at all three time points (p < 0.05), with values 8 h post-surgery being higher than preoperative values.

In the control group, a statistically significant difference in respiratory rates was observed at all three time points (p < 0.05), with values immediately post-surgery and 8 h post-surgery higher than the preoperative values. Similarly, a statistically significant difference was found in body temperature values at all three time points (p < 0.05), with values immediately post-surgery being higher than preoperative values.

### Pain

A comparison of pain values between and within the groups is presented in Table 4. A statistically significant difference was observed in the pain values immediately post-surgery and 8 h post-surgery between the groups (p < 0.05). Pain values in the control group (6.53 ± 1.36 immediately post-surgery and 6.57 ± 1.17 8 h post-surgery) were higher than those in the experimental group (3.35 ± 1.43 immediately post-surgery and 1.29 ± 1.08 8 h post-surgery).

In the experimental group, a statistically significant difference in pain values was observed at all three time points (p < 0.05). The pain values immediately post-surgery were higher than pre-surgery and 8-h post-surgery values.

In the control group, a statistically significant difference in pain values was observed at all three time points (p < 0.05). The pain values immediately post-surgery and 8 h post-surgery were higher than preoperative values.

### Anxiety

A comparison of anxiety values between the groups is presented in Table 4. A statistically significant difference in anxiety values was observed between the groups at all three time points (p < 0.05). Anxiety values were higher in the control group (60.00 ± 21.26 pre-surgery, 69.41 ± 12.56 immediately post-surgery, and 67.0 ± 11.37 8 h post-surgery) than in the experimental group (pre-surgery 45.10 ± 17.82, 24.12 ± 11.17 immediately post-surgery, and 12.35 ± 10.50 8 h post-surgery).

In the experimental group, a statistically significant difference in anxiety values was observed at all three time points (p < 0.05). Pre-surgery anxiety values were higher than immediately post-surgery and 8-h post-surgery values. Additionally, anxiety values immediately post-surgery were higher than 8 h post-surgery values.

In the control group, a statistically significant difference in anxiety values was observed at all three time points (p < 0.05). Anxiety values immediately post-surgery and 8 h post-surgery were higher than pre-surgery values.

## Discussion

This study aimed to examine the effects of watching cartoons through VR glasses before surgery on postoperative pain and anxiety levels in children undergoing tonsillectomy and adenoidectomy. The findings showed no statistically significant differences in demographic characteristics between the experimental and control groups. Age, sex, educational status, previous surgical experience, and parental age were homogeneously distributed in both groups. The absence of significant differences in variables such as children’s age and sex ensured that the effects of the VR intervention could be evaluated independently of these factors. This study contributes to existing literature by demonstrating that VR cartoon viewing can be an effective, low-cost, and noninvasive method to reduce perioperative stress markers, including systolic blood pressure, in pediatric surgical patients.

Similar studies have shown a homogeneous distribution of children’s demographic characteristics, minimizing external influences between groups and allowing a more accurate assessment of the effects of the intervention. This homogeneity enabled a more objective evaluation of the pain- and anxiety-reducing effects of the VR intervention in children. Additionally, this finding suggests that VR is applicable across a broad patient population. Consistent with previous studies [24,25], the homogeneous demographic distribution strengthens the reliability of this study.

More children in the control group than in the experimental group experienced seven to 10 infections per year (82.4% vs. 60.8%). Randomization was performed to ensure that the sociodemographic and medical characteristics of the groups were comparable. However, differences were observed between the groups regarding parental education levels and the frequency of tonsil infections in the past year. Specifically, the experimental group had a higher proportion of parents, particularly mothers, who had completed high school, whereas the children in the control group experienced more frequent tonsil infections. These differences are clearly presented to avoid potential bias in the interpretation of the results. We believe that these differences do not directly affect our aim of evaluating the effects of VR glasses on postoperative pain and anxiety. However, we acknowledge that these factors could potentially influence the findings of this study, and this possibility was considered in our interpretation.

When comparing preoperative fear, crying, and parental anxiety between the groups, the proportion of children who experienced preoperative fear was significantly higher in the experimental group than in the control group (p = 0.027). This may indicate that the children initially experienced more preoperative tension, but this anxiety was successfully alleviated by the distraction provided by the VR intervention. Furthermore, the duration of crying was significantly shorter in the experimental group (p = 0.001), supporting the notion that VR glasses are effective in reducing children’s fear and stress levels by diverting their attention (Table 3).

Regarding parental anxiety, preoperative fear rates among mothers were higher in the control group than in the experimental group (p = 0.017). This finding suggests that VR glasses not only reduced stress and fear in children but also had a calming effect on parents. Additionally, the higher education levels of mothers in the experimental group than in the control group might suggest that education and knowledge about the surgical process are linked to greater awareness and ability to manage anxiety. It is possible that mothers with higher education levels are better equipped to guide their children through medical procedures, thereby managing fear more effectively.

The lack of similar studies on crying duration indicates a need for further research on this topic. The absence of these data highlights an area to which this study contributes significantly, reinforcing the efficacy of VR glasses as a distraction technique for reducing both pre- and postoperative anxiety and stress in children.

In surgical procedures, particularly in children, anxiety and fear associated with surgery can significantly influence the perception of pain and have long-term effects on emotional and psychological well-being. Research suggests that preoperative anxiety not only intensifies the child’s pain experience but also negatively impacts postoperative recovery. High levels of anxiety before surgery are associated with greater pain perception and poorer postoperative outcomes, making it essential to address these concerns and improve recovery [3].

Today, nurses use non-pharmacological distraction techniques such as VR glasses to reduce pain and anxiety related to medical interventions. These techniques include providing information, showing cartoons, and introducing medical procedures to children. Research has demonstrated that audiovisual distraction methods are effective in managing pain and anxiety in children [23,26,27].

Nurses play a crucial role in alleviating pain and anxiety in children through therapeutic communication and interventions; however, pediatric patients often experience fear and distress in hospital settings because of medical procedures. One effective tool to address this challenge is the use of VR headsets, which, by design, completely immerse users and block their view of the external environment. This immersive quality helps to distract children from the medical interventions they are undergoing, reducing their perception of pain and anxiety. Studies have shown the efficacy of VR in pediatric care, highlighting that its ability to divert attention from the external stimuli of medical procedures significantly lowers the emotional and sensory impact on young patients. Previous studies support the notion that VR can be an effective non-pharmacological intervention, providing a calming and engaging alternative that aids in minimizing stress associated with hospital visits for children [28–30].

In our study, Cronbach’s alpha coefficient for the PMSA-C was 0.845. A high reliability coefficient indicated that the scale had good internal consistency and reliability. This result supports the reliability of our findings and is consistent with those of other studies in which the scale was used. For instance, in a study conducted by Çelik et al. [22], Cronbach’s alpha coefficients for the same scale were found to be 0.858 before surgery, 0.916 on the day of surgery, and 0.864 1 month after surgery. The consistency across studies demonstrates the reliability of the scale.

During the preoperative period, most of the children reported feeling fearful, with a higher rate observed in the experimental group (86.3% vs. 98%). This finding indicates that the children in the experimental group, who watched the *Shrek* cartoons via VR glasses, had lower pain and anxiety scores than those in the control group.

Other studies reported similar results. Dehghan et al. (2016) found that children undergoing abdominal surgery who were introduced to the surgical process through VR glasses and given a tour of the operating room had lower pain and anxiety scores than did those in the control group [31]. Ryu et al. [32] showed that using VR games in the preoperative process helped reduce anxiety in children, creating a statistically significant difference compared with the control group. Similarly, Aşkan and Sarıalioğlu [33] found that showing cartoons and musical games to children undergoing invasive procedures in the ward significantly reduced their pain and fear levels compared with those in the control group. Kuo et al. [34] also demonstrated that showing cartoons to children undergoing invasive procedures reduced their pain and fear scores.

In the present study, children who watched cartoons using VR glasses before and after surgery had significantly lower pain and anxiety scores compared with the control group (p < 0.001). Pain scores were assessed postoperatively in the recovery room and 8 h post-surgery, revealing a statistically significant difference between the groups. These findings suggest that VR glasses may effectively reduce children’s perception of pain and anxiety by diverting their attention during the perioperative period.

The findings of this study revealed that distraction and informative practices using VR glasses effectively reduced children’s anxiety and pain scores. Sociodemographic status, fear levels, and vital signs of the children assessed before the procedure were similar between the experimental and control groups in the preoperative period. Children who watched the short films had lower fear and pain scores than did those who were educated using a video explaining the surgical process. Consistent with the findings of this study, Bozkul et al. [35] found that children who watched short films experienced reduced pain and fear.

In this study, it was found that the blood pressure, pulse rate, respiration, and body temperature values of children who watched the *Shrek* cartoon with VR glasses and were comforted by their mothers (experimental group), as well as those of children who were only comforted by their mothers (control group), remained within normal limits. These findings suggest that pain and anxiety did not significantly affect vital signs in either group. This may indicate that sympathetic activity due to pain and anxiety was not elevated and that physiological changes related to surgery were well managed, consistent with the literature [36,37]. However, further studies are required to confirm this hypothesis.

Based on the findings of this study, nurses should consider using VR glasses to display cartoons as an effective method for managing pain and anxiety in hospitalized children or those undergoing surgical and interventional procedures. By employing distraction techniques, such as VR, children can shift their focus from discomfort, which may enhance cooperation during medical procedures. Previous research [37] has also supported the efficacy of VR in reducing pain and anxiety, although further studies are required to confirm these results, particularly in the surgical context.

One limitation of this study was that it only included children aged 7–12 years who underwent tonsillectomy at a single hospital, making it difficult to generalize the findings to other settings or populations. Therefore, the conclusions drawn from this study are specific to this particular study group and cannot be broadly generalized to wider populations.

## Conclusion

The findings of this study demonstrated that using VR glasses to show cartoons significantly reduced pain and anxiety levels in children undergoing tonsillectomy and adenoidectomy. Our study revealed that children in the experimental group had significantly lower pain and anxiety scores than did those in the control group. These results suggest that incorporating VR technology into pediatric surgical care can provide a non-pharmacological means to alleviate stress and discomfort and improve the overall surgical experience for children. Furthermore, watching cartoons via VR glasses had no adverse effects on physiological parameters. This reinforces the idea that VR is not only effective but also safe.

Further evidence-based research should be conducted on this intervention to determine how VR technology can be applied to various age groups or surgical procedures, investigate the long-term effects of VR glasses, and explore the impact of various VR intervention contents (e.g., educational videos and games). Furthermore, future studies should compare VR with other distraction techniques, such as music therapy, storytelling, or tablet-based games, to identify the most effective strategy for perioperative stress reduction.

## Supporting information

S1 Data Raw data for systolic blood pressure, pain, and anxiety scores of participants at all time points. (CSV)

S2 Data Participants’ sociodemographic and clinical characteristics used in the analysis. (SAV)

S1 Checklist CONSORT 2010 Checklist. (DOC)

S1 Protocol Study_Protocol_NCT_clinical_number. (PDF)
