# Supplementary material for: Effect of preoperative virtual reality cartoon viewing on postoperative pain and anxiety in children undergoing tonsillectomy and adenoidectomy: A randomized controlled trial
Source: PLoS One. 2025 Sep 11;20(9):e0331793. doi: 10.1371/journal.pone.0331793 (PMC12425227; doi:10.1371/journal.pone.0331793)
Supplement: S1 Protocol — (PDF) [file pone.0331793.s004.pdf]

ClinicalTrials.gov Protocol Registration and Results System (PRS) Receipt
Release Date: January 1, 2025

ClinicalTrials.gov ID: NCT06763276

Study Identification

Unique Protocol ID: İzmirTınaztepeÜ-B.Dizer-001

Brief Title: Preoperative VR Cartoon Viewing Reduces Postoperative Pain and Anxiety in Pediatric Tonsillectomy Patient

Official Title: The Effect of Preoperative Virtual Reality Cartoon Viewing on Postoperative Pain and Anxiety in Children Undergoing Tonsillectomy and Adenoidectomy

Secondary IDs:

Study Status

Record Verification: January 2025

Overall Status: Completed

Study Start: November 10, 2023 [Actual]

Primary Completion: June 4, 2024 [Actual]

Study Completion: July 13, 2024 [Actual]

Sponsor/Collaborators

Sponsor: Izmir Tinaztepe University

Responsible Party: Principal Investigator
Investigator: Berna Dizer [BDizer]
Official Title: Assistant professor
Affiliation: Izmir Tinaztepe University

Collaborators:

Oversight

U.S. FDA-regulated Drug: No

U.S. FDA-regulated Device: No

U.S. FDA IND/IDE: No

Human Subjects Review: Board Status: Approved
Approval Number: 230-230-14
Board Name: Uşak University Non-Interventional Research Ethics Committee
Board Affiliation: Research ethics commission
Phone: 0276 221 21 21
Email:
Address:

Uşak University Faculty of Medicine, Ankara-Izmir Road, 8th Km, 1 Eylül Campus, Uşak, Turkey

Data Monitoring: No

FDA Regulated Intervention: No

Study Description

Brief Summary: The aim of this study is to determine the effect of watching a cartoon with a virtual reality headset before surgery on the pain and anxiety levels of children aged 7-12 years.

Detailed Description: The aim of this study is to determine the effect of watching a cartoon with a virtual reality headset before surgery on the pain and anxiety levels of children aged 7-12 years. In line with this aim, children aged 7-12 who were scheduled for tonsillectomy and adenoidectomy at the Ear, Nose, and Throat Department of Uşak University Education and Research Hospital were shown a cartoon with a virtual reality headset before surgery, and their pain and anxiety levels were examined. The study was conducted as a randomized controlled experimental study at the Ear, Nose, and Throat Unit of Uşak University Education and Research Hospital. The population of the study consisted of children aged 7-12 who applied for tonsillectomy and adenoidectomy surgery at the Ear, Nose, and Throat Unit of Uşak University Education and Research Hospital. The sample of the study included a total of 102 children, with 51 in the experimental group and 51 in the control group, who met the research criteria and volunteered to participate in the study between November 10, 2023, and June 1, 2024. Data collection tools included the informed consent form, sociodemographic questionnaire, vital signs assessment form, Visual Analog Scale, and the Perioperative Multidimensional Anxiety Scale for children.

Conditions

Conditions: Tonsillectomy
Adenoidectomy

Keywords: Virtual Reality
Cartoon
Pain Level
Anxiety Level

Study Design

Study Type: Interventional

Primary Purpose: Treatment

Study Phase: N/A

Interventional Study Model: Parallel Assignment

Number of Arms: 2

Masking: Single (Participant)

Allocation: Randomized

Enrollment: 102 [Actual]

Arms and Interventions

Arms	Assigned Interventions
Experimental: Virtual Reality Group<br>A meeting was conducted with the children and their parents. Information about the content of the research was provided. Consent forms were completed by the children and their parents. Instructions on how to use the virtual reality headset were given to the children and their parents.<br><br>Mothers were asked to comfort and caress their children during the preoperative period.<br><br>The first 15-20 minutes of the cartoon were shown to the children before the surgery. After watching the cartoon, the children were sent for tonsillectomy and adenoidectomy surgery. The remainder of the cartoon was shown one hour after the surgery once the child had settled in the recovery room.<br><br>Before the tonsillectomy and adenoidectomy surgery, immediately after the child returned to the ward post-surgery, and 8 hours after the surgery:<br><br>The child's vital signs were recorded. The child's anxiety and pain scores were assessed.	Virtual Reality Group<br>In the study, a 400-gram virtual reality (VR) headset with an anti-reflective lens system was used, compatible with all earphones with screens ranging from 4.5 to 7.0 inches. The virtual reality (VR) headset, which was lightweight enough for children to wear, was supported on three sides to fit securely on the children's heads and was disinfected after each use. For the experimental group, the compartment at the front of the virtual reality (VR) headset was removed. The earphone was placed into the removed compartment, which was then reinserted. Subsequently, the YouTube virtual reality (VR) feature was activated on the phone, making it ready for viewing.
No Intervention: Control Group<br>Consent forms were completed by the children and their parents. Mothers were asked to comfort and caress their children.<br><br>Before the tonsillectomy and adenoidectomy surgery, immediately after the child returned to the ward post-surgery, and 8 hours after the surgery:<br><br>The child's vital signs were recorded. The child's anxiety and pain scores were assessed. The children in the control group were allowed to watch the cartoon via virtual reality (VR) after all data for the research had been collected to ensure ethical equality.	

Outcome Measures

Primary Outcome Measure:

1. Vital signs
   Systolic Blood Pressure (mmHg) Description: Systolic blood pressure levels measured during the perioperative period.

   [Time Frame: Preoperative, immediately postoperative, and 8 hours postoperative.]
2. Diastolic Blood Pressure (mmHg)
   Diastolic blood pressure levels will be measured to assess changes in blood pressure during the perioperative period.

   [Time Frame: Preoperative, immediately postoperative, and 8 hours postoperative]
3. Heart Rate (bpm)
   Heart rate will be measured to monitor cardiac activity during the perioperative period.

   [Time Frame: Preoperative, immediately postoperative, and 8 hours postoperative]
4. Body Temperature (°C)
   Body temperature will be measured to observe temperature changes during the perioperative period.

[Time Frame: Preoperative, immediately postoperative, and 8 hours postoperative]

5. Hemoglobin (g/dL)
   Hemoglobin levels will be measured preoperatively and within 24 hours postoperatively to evaluate changes in blood parameters during the perioperative period.

   [Time Frame: Preoperative and 24 hours postoperative]
6. Creatinine (mg/dL)
   Creatinine levels will be measured preoperatively and within 24 hours postoperatively to monitor renal function during the perioperative period.

   [Time Frame: Preoperative and 24 hours postoperative]
7. Glucose (mg/dL)
   Glucose levels will be measured preoperatively and within 24 hours postoperatively to assess perioperative glucose regulation

   [Time Frame: Preoperative and 24 hours postoperative]

Secondary Outcome Measure:

8. Secondary Outcome Measure
   Pain Score: The Visual Analog Scale (VAS) was used to measure pain intensity in children during the postoperative period following tonsillectomy and adenoidectomy. The children were assessed using the Visual Analog Scale (VAS) before surgery, immediately after returning to the ward post-surgery, and 8 hours after surgery.The Visual Analog Scale (VAS) ranges from 0 to 10, with 0 indicating no pain and 10 representing the worst possible pain. Higher scores indicate greater pain intensity.

   [Time Frame: Preoperative, immediately postoperative, 8 hours postoperative]

Other Pre-specified Outcome Measures:

9. Tertiary Outcome Measure
   Anxiety Score: The Perioperative Multidimensional Anxiety Scale for Children is a visual analog scale consisting of 5 items, each scored between 0 and 100. The scale was used to measure anxiety levels in children during the perioperative period.

   [Time Frame: Preoperative, immediately postoperative, 8 hours postoperative]

Eligibility

Minimum Age: 7 Years

Maximum Age: 12 Years

Sex: All

Gender Based: Yes

Accepts Healthy Volunteers: Yes

Criteria: Inclusion Criteria:

Child's criteria:

- Children aged between 7 and 12 years
- Ability to understand pain and anxiety scales
- Possession of communication skills
- Absence of mental issues
- Absence of physical illnesses
- Absence of chronic illnesses
- Willingness of the child and family to participate in the study
- No visual, hearing, or speech problems

Exclusion Criteria:

- Children younger than 7 years or older than 12 years
- Presence of any chronic illness that could affect pain and anxiety levels
- Presence of psychosocial issues
- Children or parents who meet the inclusion criteria but do not wish to wear the virtual reality headset (VRH) glasses
- Children who need to wear glasses other than the virtual reality headset (VRH)
- Children with hearing problems
- Children who do not wish to watch the shrek cartoon

Contacts/Locations

Central Contact Person: Berna Dizer, PhD
Telephone: 05056261042
Email: berna_kizilkaya@yahoo.com

Central Contact Backup: Berna Dizer, PhD
Telephone: 05056261042
Email: berna_kizilkaya@yahoo.com

Study Officials:

Locations: **Turkey**
Izmir Tinaztepe University
Izmir, Buca, Turkey, 35400
Contact: Berna Dizer 05056261042 berna_kizilkaya@yahoo.com

IPDSharing

Plan to Share IPD: No

References

Citations:

Links:

Available IPD/Information:
